# Dysfunction of Sister Chromatids Separation Promotes Progression of Hepatocellular Carcinoma According to Analysis of Gene Expression Profiling

**DOI:** 10.3389/fphys.2018.01019

**Published:** 2018-07-27

**Authors:** Baozhen Sun, Guibo Lin, Degang Ji, Shuo Li, Guonan Chi, Xingyi Jin

**Affiliations:** ^1^Department of Hepatopancreatobiliary, China-Japan Union Hospital of Jilin University, Changchun, China; ^2^First Department of Neurosurgery, China-Japan Union Hospital of Jilin University, Changchun, China

**Keywords:** separation of sister chromatids, HCC, biomarker, SAM, GSEA

## Abstract

Despite studying the various molecular mechanisms of hepatocellular carcinoma (HCC), effective drugs and biomarkers in HCC therapy are still scarce. The present study was designed to investigate dysregulated pathways, novel biomarkers and therapeutic targets for HCC. The gene expression dataset of GSE14520, which included 362 tumor and their paired non-tumor tissues of HCC, was extracted for processing by the Robust multi-array average (RMA) algorithm in the R environment. SAM methods were leveraged to identify differentially expressed genes (DEGs). Functional analysis of DEGs was performed using DAVID. The GeneMania and Cytohubba were used to construct the PPI network. To avoid individual bias, GSEA and survival analysis were employed to verify the results. The results of these analyses indicated that separation of sister chromatids was the most aberrant phase in the progression of HCC, and the most frequently involved genes, EZH2, GINS1, TPX2, CENPF, and BUB1B, require further study to be used as drug targets or biomarkers in diagnosis and treatment of HCC.

## Introduction

Hepatocellular carcinoma (HCC) is the third-leading cause of cancer-related deaths worldwide and its incidence continues to rise (Mittal and El-Serag, 2013). It mainly arise from hepatitis B virus (HBV) or hepatitis C virus (HCV) infections, and patients with cirrhosis have more opportunities to get HCC (El-Serag, 2002; Umemura et al., 2009). The limited knowledge on the molecular mechanisms of HCC contribute to poor prognosis and ineffective therapy, which leaves liver transplantation as the best choice of management (Ho et al., 2015; Turato et al., 2017). However, recurrence following transplantation is also associated with an unfavorable prognosis (Spinzi and Paggi, 2008; Sposito et al., 2013). Moreover, surgical intervention is ineffective in patients diagnosed at advanced stages of HCC (Rich et al., 2017). Therefore, new therapies for HCC are direly needed.

Although some molecular events that facilitate the progression to HCC have been investigated, the effective drug targets and potential biomarkers for early treatment and diagnosis of HCC are still unclear (Blum, 2005; Cha and Dematteo, 2005). Therefore, identifying the dysregulated pathways and hub genes involved in this process would allow us to identify patients with HCC as early as possible. Findings from previous studies that have focused on this area are limited due to small sample sizes, resulting in an incomplete understanding of HCC (Jia et al., 2007; Lin et al., 2014; Yin et al., 2017).

In this study, we extracted the gene expression profiles from the GEO database of 362 HBV-related HCC tumors and their paired non-tumor tissues which are mostly accompany with liver cirrhosis. The significance analysis of microarrays (SAMs) algorithm was used to screen the differential expressed genes (DEG), which was performed for pathway enrichment and generation of PPI network. After that, hub genes were identified by GeneMANIA and CytoHubba in Cytoscape. Furthermore, we performed a gene set enrichment analysis (GSEA), which evaluates microarray data at the level of gene sets, to overcome the limitation of individual gene analysis. In the meantime, survival analysis was leveraged, using the TCGA database, to assess the risks of hub gene expression. Finally, five most significantly hub genes were verified by qPCR and IHC in human HCC to confirm the results.

## Results

### Microarray Data Collection and Processing

The BioConductor package, Simpleaffy, was used for quality control and normalization of the microarray raw files (**Supplementary Figure S1**).

### Identification of DEGs

**FIGURE 1:**
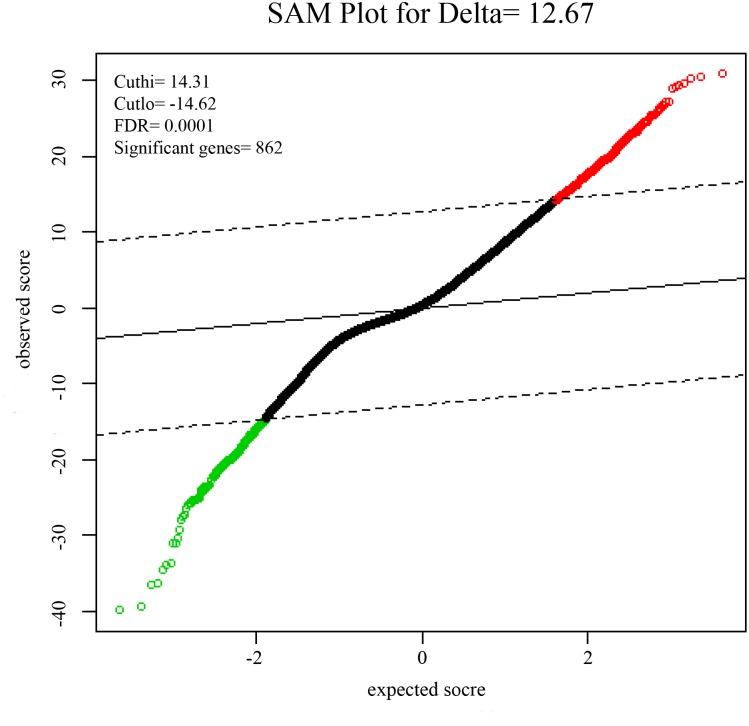
Significance analysis of microarrays (SAMs) plot comparing gene expression of HCC. 553 up-regulated genes are colored in red, 309 down-regulated genes are colored in green.

To identify DEGs from the tumor and non-tumor tissue of HCC patients, we used the SAM method at the delta 14.31, with the FDR < 0.1%. A total of 862 genes were identified as DEGs, including 553 up-regulated and 309 down-regulated genes (**Figure 1**).

### Functional Analysis of DEGs

**FIGURE 2:**
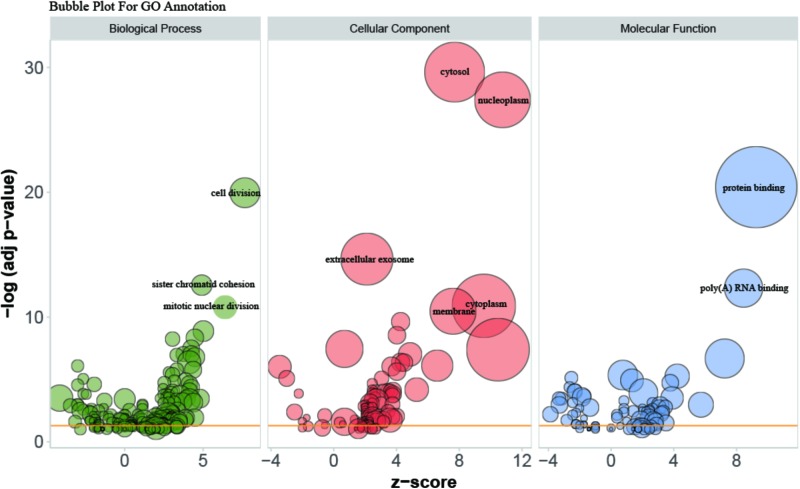
Bubble plot for visualizing GO Annotation. Term are shown at –log (adj *p*-value) cut off >10. The z-score predicts existence of a bias in gene regulation.

**FIGURE 3:**
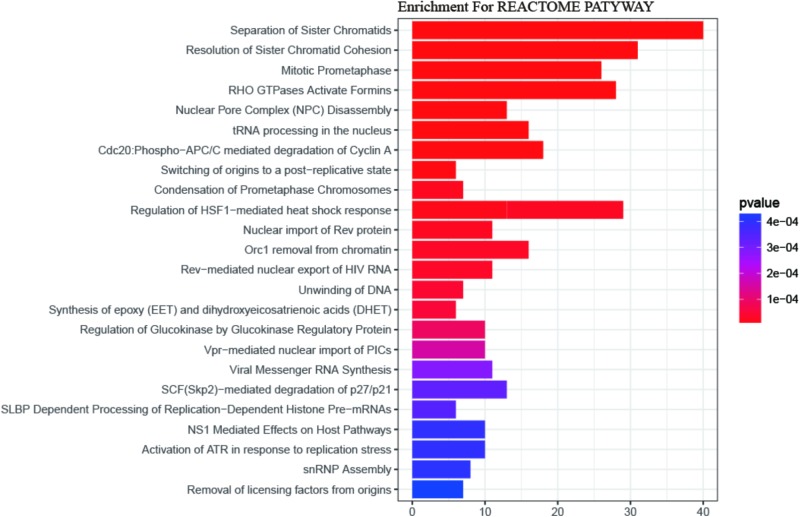
The REACTOME pathway enrichment analysis of DEGs. 24 statistically significant pathways are listed and their colors are shown by *p*-values.

Gene Ontology (GO) enrichment analysis revealed that GO terms were most significantly enriched in cell division (*p* = 1.09E-20), sister chromatid cohesion (*p* = 2.79E-13), mitotic nuclear division (*p* = 1.62E-11) and DNA replication (*p* = 1.34E-09; **Figure 2**). Reactome Pathway enrichment analysis showed that the separation of sister chromatids was the most significantly affected phase in HCC (**Figure 3**), which was in accordance with the results of the GO enrichment analysis.

### PPI Network Analysis of DEGs

**FIGURE 4:**
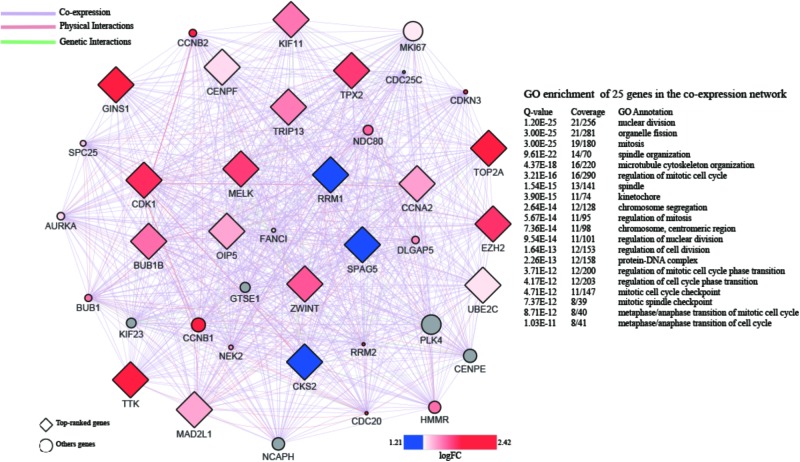
PPI network of 20 top-ranked DEGs. The hub genes are screened by cytoHubba and colored by their logFC.

A PPI network with 873 nodes and 84,272 edges was generated via the GeneMANIA plugin, around the DEGs. A global metric was utilized in the determination of hub proteins, through the Cytohubba plugin. Following this, the relationship between the 20 top-ranked proteins was mapped, based on the MCC as shown in **Figure 4**. The majority of these were cell division-related genes, such as TOP2A, GINS1, BUB1B, TPX2, and CENPF. The 20 top-ranked proteins were all up-regulated DEGs.

### GSEA and Leading-Edge Analysis

**FIGURE 5:**
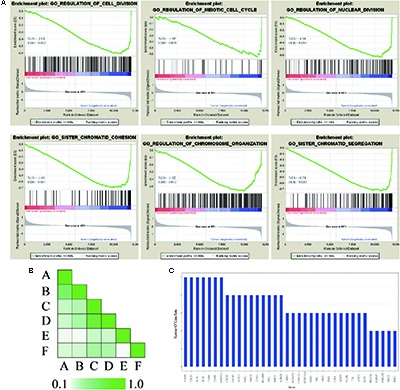
GSEA of gene expression profiling of HCC. **(A)** GO enrichment plot of six cell division related gene sets. The normalized enrichment score (NES) and the false discovery rates (FDR) are indicated for each gene set. Each bar at the bottom of plot represents a member gene of the respective gene set. **(B)** Leading edge analysis among gene sets. Color intensity is used to show the overlap between subsets, the darker the color, the greater the overlap between the subsets. Specifically, the intensity of the cell for sets A and B corresponds to an X/Y ratio where X is the number of leading edge genes from set A and Y is the union of leading edge genes in sets A and B. **(C)** The bar graph shows each gene and the number of subsets in which it appears.

In order to further confirm the molecular mechanisms of HCC in the whole transcriptome, GSEA of the gene expression profile data from 362 tumor and paired non-tumor tissue of HCC patients was performed, based on the GO biological process. The results revealed that the most significant biological processes that were enriched were cell division-related processes, including GO_REGULATION_OF_ CELL_DIVISION (FDR = 0.052), GO_REGULATION_ OF_MEIOTIC _CELL_CYCLE (FDR = 0.050), GO_REGULATION_ OF_NUCLEAR_DIVISION (FDR = 0.051), GO_SISTER_ CHROMATID_COHESION (FDR = 0.051), GO_REGULATION_OF_ CHROMOSOME_ORGANIZATION (FDR = 0.043), and GO_SISTER_ CHROMATID_SEGREGATION (FDR = 0.033), which are shown in **Figure 5A**. Leading-edge analysis was used to find the hub genes which appeared frequently in related gene sets, and the results showed that AURKB appeared in five gene sets, while BUB1B, CDC20, FBXO5, DLGAP5, ESPL1, BIRC5, BUB3, BUB1, CENPE, CENPF, and MAD2L1 appeared in four gene sets (**Figures 5B,C**).

### Kaplan–Meier Survival Analysis

**FIGURE 6:**
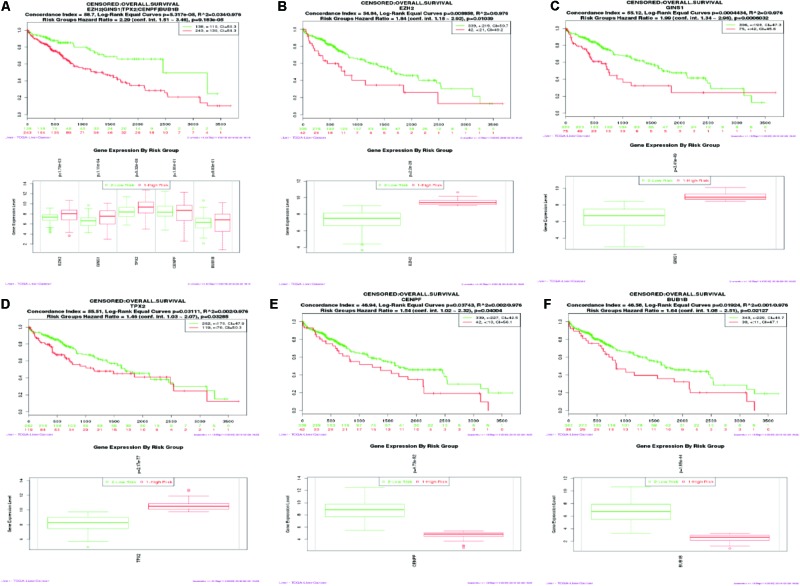
Kaplan–Meier survival curve of 422 TCGA liver cancer samples using the SurvExpress database, based on the low or high risk for a poor outcome. Censoring samples are marked with “+.” The Horizontal axis represents time to event and vertical axis represents percentage. High- and low-risk groups are labeled with red and green curves, respectively. **(A)** High expression of five hub genes is correlated with high risk, poor prognosis and shorter overall survival time. **(B–F)** Survival curve of five hub genes respectively.

SurvExpress was engaged to explore the relationship between the hub genes and the survival of HCC patients in silico. Finally, survival analysis, based on clinical information from the TCGA-liver cancer datasets, revealed that the high expression of EZH2, GINS1, and TPX2 correlated positively with higher risk, CENPF and BUB1B were quite the contrary (**Figure 6**).

### Validation of Hub Genes by qPCR in Human HCC

**FIGURE 7:**
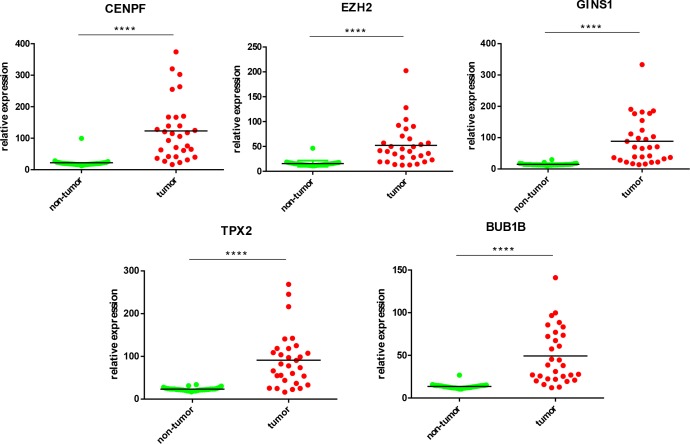
Validation of five hub genes by qPCR. HCC sample (red) and their paired non-tumor tissue (green) were validated by qPCR, ^∗∗∗∗^*P* < 0.0001.

Five most significantly hub genes, EZH2, GINS1, TPX2, CENPF, and BUB1B, were successfully validated by qPCR in 30 paired human HCC tissues which have no difference with the analysis results of gene expression profiling (**Figure 7**).

### Immunohistochemistry

**FIGURE 8:**
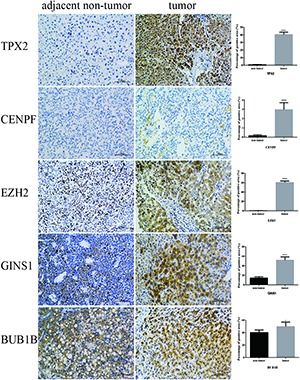
Validation of five hub genes by IHC. Representative photomicrographs of immunohistochemical detection and measurement of five hub genes by Image Pro Plus 6.0 between 12 paired tumor and adjacent non-tumor tissues, ^∗^*P* < 0.05 and ^∗∗∗∗^*P* < 0.0001 (magnification 200×).

IHC was employed to validate the results from bioinformation analysis, which revealed the strong expression of five hub genes in HCC vs. the control group. However, CENPF and BUB1B were not outstandingly in our result, the individual difference may affect the outcome to some extent (**Figure 8**).

## Discussion

In the present study, we explored the molecular mechanisms of HCC between tumor and non-tumor tissues, using bioinformatics analysis. Our results indicated that the separation of sister chromatids was the most significantly dysregulated pathway during the transition from cirrhosis to HCC, with the up-regulation of 12 hub genes.

One of the most miraculous events in the human cell cycle is the concurrent separation of 46 pairs of sister chromatids. Since this irreversible separation is highly monitored and regulated, neither damage to the genome, nor errors in chromosome alignment, can be easily rectified after separation (Nasmyth, 1999). The initiation of chromosomal segregation was supervised by the spindle assembly checkpoint (SAC), which ensures the genomic stability during mitosis (Dai, 2009). Defects in sister chromatid segregation could lead to aneuploidy (Panigrahi and Pati, 2009) and promote chromosome instability (CIN) during mitosis (Schvartzman et al., 2010). In addition, CIN may contribute to tumor initiation and/or progression, which has been demonstrated in cell lines (Zhang et al., 2004; Vader and Lens, 2008), mice models (Bernal et al., 2002; Dawlaty et al., 2008), and human tumors (Kronenwett et al., 2004; Carter et al., 2006). In the Sgo1^±^ mouse model, mitotic error-induced CIN was shown to be an important early event in HCC development (Kronenwett et al., 2004). Furthermore, 120 HCC with 195 markers (Nagai et al., 1997), and 48 HCC with 275 chromosomal markers (Boige et al., 1997), indicated that CIN appears widely in HCC. Aneuploidy is a major manifestation of CIN and is seen in over 75% of cancers, and is also considered essential for tumorigenesis, by some biologists (Duesberg and Li, 2003). Therefore, dysregulation of sister chromatids separation might contribute to the initiation and progression of human HCC.

Based on the results of the PPI analysis by GeneMania and Cytohubba, 20 top-ranked proteins from 862 DEGs are thought to participate in the core pathway of HCC, such as TOP2, GINS1, EZH2, TTK2, CDK1, BUB1B, TPX2, CENPF, and MAD2L1. Furthermore, the high expression of GINS1, EZH2, and TPX2, correlates with high-risk in HCC, as confirmed by the survival analysis. BUB1B, TPX2, and CENPF appeared most often in related gene sets, which were demonstrated by GSEA at the level of gene sets. Therefore, EZH2, GINS1, TPX2, CENPF, and BUB1B are thought to be hub genes in HCC and are discussed below.

Epigenetic regulation of gene expression, particularly hypermethylation, plays an important role in tumorigenesis (Jones and Baylin, 2002). EZH2 is the enzymatic subunit of Polycomb repressive complex 2 (PRC2), which methylates H3K27, resulting in silence of the associated tumor suppressor genes (Margueron and Reinberg, 2011; Momparler et al., 2012). EZH2 was up-regulated and expressed in many solid cancers, and YY1 can recruit EZH2 and suppress NFkB function in hepatitis B virus-dependent HCC (Chase and Cross, 2011). Additionally, EZH2 is clinically associated with tumor progression and multiple metastatic features, and epigenetically restrained a subset of miRNA in human HCC (Au et al., 2012). Thus, EZH2 may be regarded as a potential therapeutic target, and a few of compounds have been already investigated as inhibitors of EZH2, in pre-clinical studies (Knutson et al., 2012, 2013; McCabe et al., 2012; Qi et al., 2012; Kim et al., 2013).

GINS1/PSF1 is a subunit of the GINS complex, which is involved in the DNA replication fork and the initiation of chromosome replication (Labib and Gambus, 2007). Research suggests that GINS1 and/or other GINS complex subunits are upregulated in some types of cancers and possess some tumorigenic characteristics (Hokka et al., 2013; Zhang et al., 2015; Zhou et al., 2015). GINS1 is expressed at high levels in HCC tissues, which is associated with more aggressive tumors and worse prognosis. Moreover, in a mouse xenograft model, high levels of GINS1 expression correspond to high proliferative activity, transplantation potential, and metastatic capability (Nagahama et al., 2010). In contrast, knockdown of GINS1 expression led to inhibited tumor growth by disrupting DNA replication and chromosomal segregation, and promoted apoptosis, particularly early apoptosis (Nagahama et al., 2010; Zhou et al., 2015). These findings may make GINS1 a potential theranostic target in the future.

For many years, the function of TPX2 has been studied in mitosis and spindle assembly because of the chromatin-mediated TPX2/Importinα-β/Ran signal and its control of Aurora A kinase (Asteriti et al., 2010). The location of TPX2 is at the long arm of chromosome 20, at position 20q11, which is often amplified in HCC and other tumors (Knuutila et al., 1998; Hodgson et al., 2003; Scotto et al., 2008; Beroukhim et al., 2010). The elevated TPX2 expression results in dysregulation of spindle formation and balanced chromosome segregation, by over activation of Aurora-A, which could lead to unscheduled phosphorylation of downstream targets. TPX2 knockdown inhibits cell proliferation and AKT signaling, and decreases the MMP2 and MMP9 expression in HCC cell lines (Liu et al., 2014). Clinical sample analysis also indicates that TPX2 expression is associated with the tumor–node–metastasis stage, tumor numbers, and tumor differentiation in the HCC tissues (Liu et al., 2015). Moreover, TPX2 inactivation experiments indicated anti-proliferative effects in cancer cells, suggesting the potential value of TPX2 as an anti-cancer target (Fenner et al., 2005; Warner et al., 2009; Li et al., 2010).

CENPF is a large coiled-coil protein whose expression and subcellular localization was cell cycle-dependent, and undergoes rapid degradation at the end of cell division. It is reported that CENPF plays a major role in kinetochore assembly, regulation of chromosome segregation, and control of SAC activity. CENPF may be a potential proliferation marker in the clinical diagnosis of HCC (Ma et al., 2006). CENPF is overexpressed in HCC (Kim et al., 2012) and other tumors (Varis et al., 2006). Additionally, the up-regulated CENPF expression has been shown to contribute to the proliferation of HCC rather than acting as a trigger for malignant cell growth. Consequently, CENPF could be an indicator of tumorigenesis, especially at early stages of HCC (Zhang et al., 2001).

BUB1B is a key component in the SAC protein family, which has been proven to be involved and upregulated in multiple human cancers (Seike et al., 2002; Shichiri et al., 2002; Gupta et al., 2003; Yamamoto et al., 2007; Fu et al., 2016). In mitosis, BUB1B accumulates cyclin B in G2 phase, by binding to CDC20 to inhibit APC/C activity and prolonging the checkpoint signaling by kinase activity at kinetochores (Malureanu et al., 2009). A human study reported that the role of BUB1B was to facilitate accurate chromosome segregation and maintain chromosomal stability, to suppress cancer (Hanks et al., 2004). However, the phosphorylated BUB1B, which is tightly regulated through its own activation and subcellular localization (Bin et al., 1998; Li et al., 1999), was elevated in the SV40 Tag-derived prostate cancer models (Guo et al., 2006). The contradiction of BUB1B, between its role in suppressing cancer and upregulating cancers, is demonstrated in varying reports of cancer-associated missense and nonsense mutations in BUB1B, in several cancers (Cahill et al., 1998, 1999; Myrie et al., 2000; Saeki et al., 2002). However, the true mechanism of BUB1B in cancers remains to be elucidated.

In conclusion, we collectively analyzed the molecular mechanisms of human HCC through interpretation of the functions and PPI network of DEGs, which were confirmed by GSEA and survival analysis. In doing so, we ascertained the molecular genetic differences between tumor and non-tumor tissues of HCC, which suggest that separation of sister chromatids may have the most important influence on initiation and progression of human HCC. Errors in this process contribute to CIN and aneuploidy which were thought to be responsible for tumorigenic progression in human cells. The hub genes we found may be useful as biomarkers for diagnosis and prognosis or in tailoring treatment in human HCC. Finally, additional studies are needed to confirm the findings of these experiments.

## Materials and Methods

### Microarray Data Collection and Processing

The gene expression dataset of GSE14520 was obtained from NCBI GEO database^1^ which is based on the Affymetrix Human Genome U133A 2.0 Array. 362 tumor and paired non-tumor tissues of HCC patients were collected for analysis of genome microarrays. The detail of every HCC patient was shown in **Supplementary Table S1**. The Simpleaffy package was used to read the raw data and perform quality control and normalization by Robust multi-array average (RMA) algorithm in the R environment (Wilson and Miller, 2005). The mean gene expression was considered in multiple probe sets with one name.

### Identification of Differentially Expressed Genes (DEGs)

The DEGs in tumor and non-tumor tissues of HCC patients were determined using SAM (Grace and Nacheva, 2012). We used two class unpaired comparison analysis with *t*-statistics, and permutations of 10^3^. SAM uses permutations of repeated measurements to estimate the percentage of genes identified by chance, and the false discovery rate (FDR).

### Functional Analysis of DEGs

Gene Ontology (GO) and Reactome Pathway enrichment analyses were performed using DAVID to explore the biological processes and signaling pathways in which the DEGs were involved (Ashburner et al., 2000; Huang et al., 2009; Fabregat et al., 2018). The enrichment results were visualized in a new R visualization package called GOPlot (Walter et al., 2015).

### PPI Network Analysis of DEGs and the Screening of Hub Proteins

A PPI network analysis was performed to evaluate physical relationships between the proteins encoded by the DEGs. The GeneMania (Montojo et al., 2010) and Cytohubba (Chin et al., 2014) were used to construct the PPI network based on co-expression, physical interactions, and genetic interactions.

### Gene Set Enrichment Analysis (GSEA) and Leading-Edge Analysis

To further investigate the biological characteristics of HCC, we performed GSEA assay in non-tumor and tumor groups with permutations of 10^4^ in the GO biological process, from the Molecular Signature Database (MSigDB). A leading-edge analysis was performed to elucidate hub genes of HCC according to the results of Reactome Pathway enrichment (Subramanian et al., 2005).

### Kaplan–Meier Survival Analysis

Kaplan–Meier analysis was performed with the online multi-cancer biomarker validation tool, SurvExpress, in the TCGA-liver cancer datasets containing 422 samples, using the hub genes as an input (Aguirre-Gamboa et al., 2013).

### Sample Collection

Thirty HBV-related HCC and their paired non-tumor tissues were collected from August 2014 to December 2017 at China-Japan Union Hospital of Jilin University. All the specimens were dealing with liquid nitrogen after surgical resection and stored at -80°C. Three independent pathologists made the decision about the diagnosis of HCC and assessed the samples with HE staining.

### Quantitative RT-PCR

Total RNA of 30 HCC samples was extracted using Trizol (Invitrogen) as described everywhere, qRT-PCR was performed by One-Step qPCR Kit (Invitrogen) and CFX Connect^TM^ Real-Time System (BIO-RAD) following manufacturer’s instructions. The data of qPCR were processed by ΔΔCt method with normalizing to GAPDH as the reference gene. The sequence of primers was shown in **Supplementary Table S2**.

### Immunohistochemistry

The 4 μm thick sections cut from formalin-fixed, paraffin-embedded HCC tissue were used for IHC of hub genes as described before (Ho et al., 2012). The primary antibodies against EZH2 (No. 191080, Abcam), BUB1B (No. 183496, Abcam), CENPF (No. 223847, Abcam), TPX2 (11741-1-AP, Proteintech) and GINS1 (No. D161403, Sangon Biotech) were employed for IHC. Image Pro Plus 6.0 (Media Cybernetics, Bethesda, MD, United States) was employed to measure the positive area of hub genes for quantitative analysis.

### Statistical Analysis

All data are shown as mean ± SDs except for otherwise indicated. Significance was determined with two-tailed *t*-test when comparing the variance from HCC to the adjacent non-tumor tissue. GraphPad Prism 6 software (GraphPad Software, La Jolla, CA, United States) was used for analysis. A *P* < 0.05 is considered significant.

## Ethics Statement

This study was carried out in accordance with the recommendations of CIOMS. The protocol was approved by the institutional review boards of the China-Japan Union Hospital of Jilin University. All subjects gave written informed consent in accordance with the Declaration of Helsinki.

## Author Contributions

XJ conceived and designed the study. BS collected, analyzed the data, and wrote the manuscript. GL, DJ, SL, and GC revised the manuscript. All authors read and approved the manuscript.

## Conflict of Interest Statement

The authors declare that the research was conducted in the absence of any commercial or financial relationships that could be construed as a potential conflict of interest.

## Footnotes

1 http://www.ncbi.nlm.nih.gov/geo/

## Supplementary Material

FIGURE S1 **(A)** Data before normalization. **(B)** Data after normalization.

TABLE S1 Samples detail.

TABLE S1 qPCR primers used for tissues specific expression analysis.
