# Supplementary figures and images for: Dysfunction of Sister Chromatids Separation Promotes Progression of Hepatocellular Carcinoma According to Analysis of Gene Expression Profiling
Source: Front Physiol. 2018 Jul 27;9:1019. doi: 10.3389/fphys.2018.01019 (PMC6072861; doi:10.3389/fphys.2018.01019)

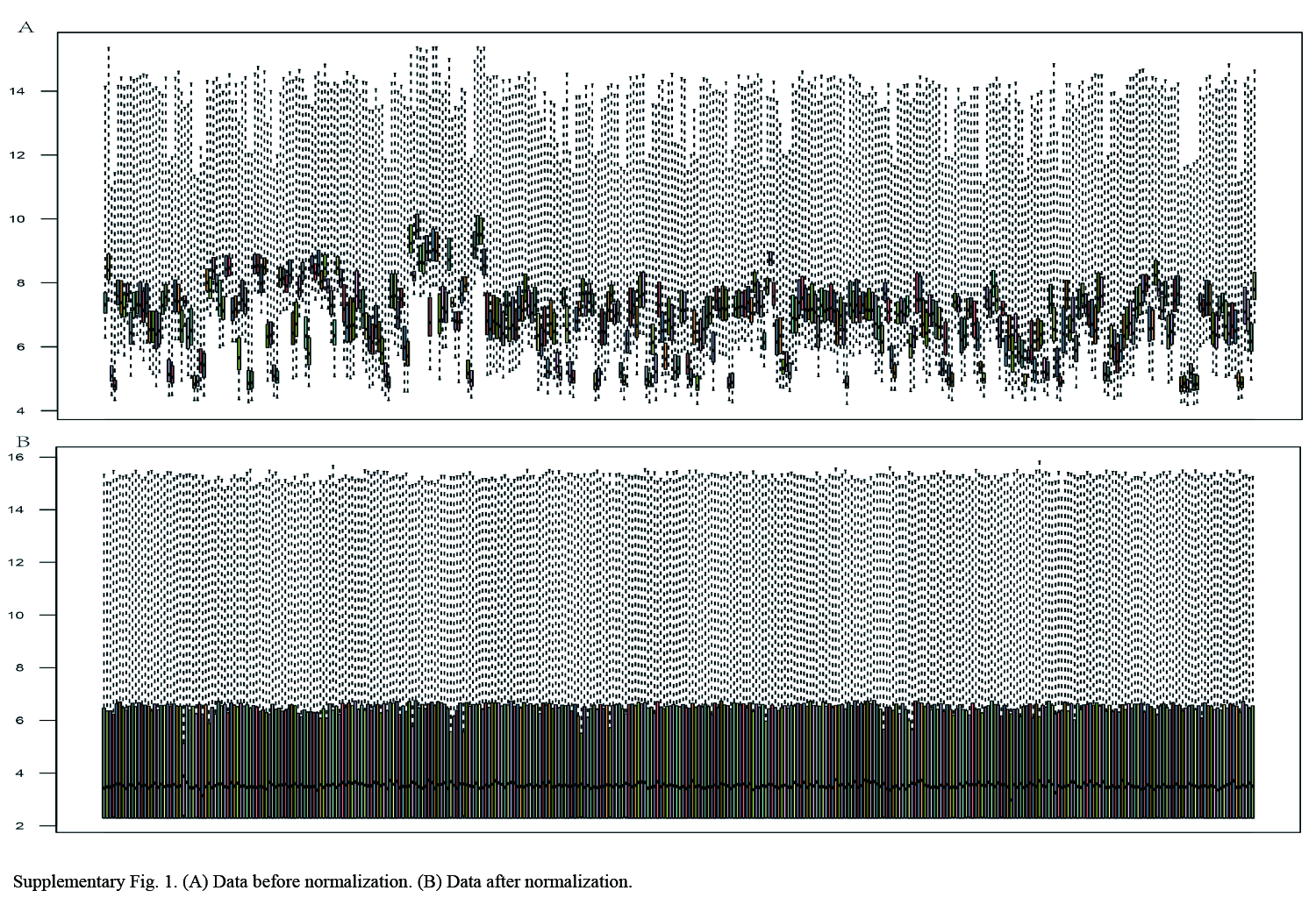

Supplement: FIGURE S1 — (A) Data before normalization. (B) Data after normalization. [file Image_1.TIF]
