# Supplementary material for: Dysfunction of Sister Chromatids Separation Promotes Progression of Hepatocellular Carcinoma According to Analysis of Gene Expression Profiling
Source: Front Physiol. 2018 Jul 27;9:1019. doi: 10.3389/fphys.2018.01019 (PMC6072861; doi:10.3389/fphys.2018.01019)
Supplement: TABLE S1 — qPCR primers used for tissues specific expression analysis. [file Table_2.PDF]

Supplementary Table 2. qPCR primers used for tissue specific expression analysis.

Target gene		Primer sequence (5' to 3')	Tm (℃)	Product size (bp)
CENPF	Forward	CTCTCCCGTCAACAGCGTTC	62.8	102
	Reverse	CTCTCCCGTCAACAGCGTTC	60.7	
EZH2	Forward	GTACACGGGGATAGAGAATGTGG	61.8	176
	Reverse	GGTGGGCGGCTTTCTTTATCA	62.7	
GINS1	Forward	ACGAGGATGGACTCAGACAAG	60.9	153
	Reverse	TGCAGCGTCGATTTCTTAACA	60.3	
TPX2	Forward	TCCTGCCCGAGTGACTAAGG	62.8	144
	Reverse	CTGTTAGGGGTTCGTTTATGGAA	60.0	
BUB1B	Forward	TAGGGCGTTTATGCAATGAGC	60.8	148
	Reverse	TCCTGAAATATCGCATCTGCTTT	60.1	
